# Using structural knowledge in the protein data bank to inform the search for potential host-microbe protein interactions in sequence space: application to *Mycobacterium tuberculosis*

**DOI:** 10.1186/s12859-017-1550-y

**Published:** 2017-04-04

**Authors:** Gaurang Mahajan, Shekhar C. Mande

**Affiliations:** 1 National Centre for Cell Science, Ganeshkhind, Pune, 411 007 India; 2 Indian Institute of Science Education and Research, Pashan, Pune, 411 008 India

**Keywords:** Protein-protein interactions, Host-pathogen interactions, Domain-domain interactions, Local sequence alignment

## Abstract

**Background:**

A comprehensive map of the human*-M. tuberculosis* (MTB) protein interactome would help fill the gaps in our understanding of the disease, and computational prediction can aid and complement experimental studies towards this end. Several sequence-based *in silico* approaches tap the existing data on experimentally validated protein-protein interactions (PPIs); these PPIs serve as templates from which novel interactions between pathogen and host are inferred. Such comparative approaches typically make use of local sequence alignment, which, in the absence of structural details about the interfaces mediating the template interactions, could lead to incorrect inferences, particularly when multi-domain proteins are involved.

**Results:**

We propose leveraging the domain-domain interaction (DDI) information in PDB complexes to score and prioritize candidate PPIs between host and pathogen proteomes based on targeted sequence-level comparisons. Our method picks out a small set of human-MTB protein pairs as candidates for physical interactions, and the use of functional meta-data suggests that some of them could contribute to the in vivo molecular cross-talk between pathogen and host that regulates the course of the infection. Further, we present numerical data for Pfam domain families that highlights interaction specificity on the domain level. Not every instance of a pair of domains, for which interaction evidence has been found in a few instances (i.e. structures), is likely to functionally interact. Our sorting approach scores candidates according to how “distant” they are in sequence space from known examples of DDIs (templates). Thus, it provides a natural way to deal with the heterogeneity in domain-level interactions.

**Conclusions:**

Our method represents a more informed application of local alignment to the sequence-based search for potential human-microbial interactions that uses available PPI data as a prior. Our approach is somewhat limited in its sensitivity by the restricted size and diversity of the template dataset, but, given the rapid accumulation of solved protein complex structures, its scope and utility are expected to keep steadily improving.

**Electronic supplementary material:**

The online version of this article (doi:10.1186/s12859-017-1550-y) contains supplementary material, which is available to authorized users.

## Background

Tuberculosis (TB) continues to pose a serious global health problem [1, 2]. The widespread prevalence of latent as well as active forms of TB disease, and the emerging threat of multi/extremely drug-resistant strains of pathogenic *Mycobacterium tuberculosis* (MTB), the underlying causative agent, present scientific and strategic challenges [3–6]. Overcoming this menace will depend, in part, on a comprehensive understanding of the molecular crosstalk between the pathogen and its human host on the cellular level at different stages of the disease [4]. Dissecting the tug of war between the invading bacterium and the phagocytic host cell that internalizes it will require mapping out the complex web of interactions between MTB virulence factors and the host cell signaling network that is engaged during infection. These protein-protein interactions (PPIs) could, on the one hand, represent the active manipulation of the host cell machinery by the pathogen, and on the other, reveal the defensive responses mounted by the host in an attempt to clear out the invader [7, 8].

Multiple changes are known to occur in the physiology of the macrophage following phagocytosis of virulent MTB [7–9]. These include disrupted trafficking and the arrest of phagosome-lysosome fusion [5, 8], inhibition of apoptotic and autophagic pathways [10–12], perturbed mitochondrial function [13], increased rendoplasmic reticulum stress [14], enhanced lipid production [15, 16], and on a broader scale, granuloma formation [17, 18], all of which contribute to pathogen survival inside the host. Another dimension of complexity has been added by the recent observation that the bacterium might be actively rupturing the phagosomal membrane to escape into the cytosol, leading to increased toxicity and necrotic cell death [19]. This extensive remodeling on the host side stems from secreted virulence factors as well as proteins associated with the complex mycobacterial cell wall with direct access to the exterior. In addition, a contribution from cytosolic MTB proteins, released by the lysis of some bacterial cells inside the phagocyte, is also possible.

Low throughput experimental studies have so far uncovered and characterized around 40 binary protein-protein interactions between MTB and human, and these have helped shed some light on the pathophysiology of the disease [20]. A recent attempt to expand this interaction network harnessed the yeast two-hybrid assay (Y2H) to map out, on the genome-wide scale, interactions between a large set of human ORFs and a filtered set of MTB ORFs having possible involvement in the infection process [21]. This experimental study found evidence for ~ 50 novel possible interactions in vitro, and detailed follow-up investigation of one novel interaction, between EsxH and the host ESCRT complex, suggested a role for this interaction *in vivo* in disrupting endosomal trafficking which in turn promotes bacterial survival. One limitation of such a high-throughput screening approach is that, possibility of false detections (false positives/negatives) cannot be ruled out. Estimates suggest that Y2H has a sensitivity of only about 20% [22], and, as admitted in [21], several known interactions could not be detected by their high-throughput experimental screen. These results taken together suggest that there is still scope, and a need, for more studies that can map out other as-yet unknown human-MTB interactions and contribute to a more complete picture of the host-pathogen interactome. Computational methods can complement and aid experimental approaches by helping to predict, or prioritize, potential interactions which could guide wet lab studies. Indeed, bioinformatic, or in silico, prediction of human-microbial PPIs has emerged as an active area of research in recent years [23, 24].

Several computational approaches to predicting PPIs, both within and between species, are based on the use of sequence information of the participating proteins [23–27]. These approaches are computationally efficient, requiring only the use of heuristic methods for sequence alignment, and are amenable to automation, which make sequence-based methods suitable for making large scale predictions on the whole-genome level. In contrast, structure-based approaches involve homology modeling based on known complex structures, possibly followed by molecular dynamics simulations, and are computatonally intensive in general [28, 29]. Thus, sequence based methods can serve as a preliminary step to narrow down the space of all possible host-pathogen protein pairs to a more manageable number and help prioritize candidate interactions, which could subsequently be analyzed in more detail through structure-based modeling and/or empirical validation. This paper proposes an improved sequence-based methodology to identify a small set of plausible PPIs between host and microbe proteins, starting from the much larger set of possible pairings encompassing the full proteomes of the two species in question.

**Fig. 1:**
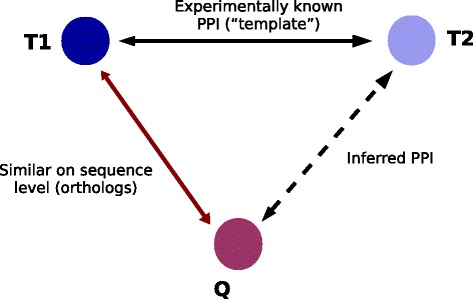
General idea behind sequence-based PPI prediction. These approaches make use of homology relationships and knowledge about protein interaction networks. An interaction is proposed to occur between the query protein Q and the protein T2, if Q shares similarity with the protein T1 on the sequence level and T1 is experimentally known to interact with T2

Sequence-based methods, in general, harness the “universe” of experimentally known PPIs, both within and across genomes, which serve as templates to search against [25–27, 30]. To ascertain whether a query pair of proteins (A, B) might interact, each protein is compared to all the proteins occurring in the template set. If the template dataset contains an interacting pair A’-B’ such that A’ and B’ are closely related to A and B respectively, then the template interaction is “carried over”, and a candidate interaction is proposed to occur between A and B. This idea is schematically depicted in Fig. 1. Several methods have been proposed in the past based on the above logic, and they differ in the exact details of the way the similarity between proteins is assessed on the sequence level. Nonetheless, they all are based on some version of local sequence alignment, e.g. using BLAST, for detecting homology relationships between proteins [25–27, 31–35].

**Fig. 2:**
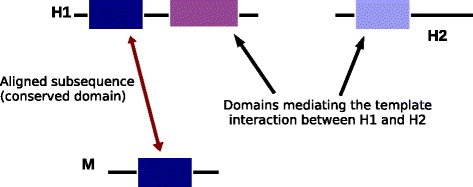
Local alignment-based comparison of protein sequences could lead to erroneous inferences when applied to the prediction of novel PPIs. In this example, the query microbial protein M shares a subsequence (functional domain) with the host protein H1. However, the template interaction between proteins H1 and H2 is mediated by a different domain. If this piece of information is unavailable, a spurious interaction between M and H2 may be inferred

BLAST is an example of heuristic local alignment [36], and essentially looks for regions of sequence similarity in the query and template proteins which could be embedded within longer full-length protein sequences, and represent a conserved functional domain, say. While this is well suited to uncovering evolutionarily conserved functions between proteins that can be quite dissimilar overall, local alignment-based search comes with a potential caveat when applied to the problem of interaction prediction, especially with multi-domain proteins. To see this, one can imagine a situation such as the one depicted in Fig. 2. The query protein M shares a conserved region (e.g. a functional domain) with the protein T that participates in a template PPI, following from local alignment-based search. However, the part of T actually involved in the template PPI is distinct from the aligned region, and may correspond to a different domain. As a consequence, labeling M as being similar to T based on the E-value or percentage identity of the aligned stretch might lead to spurious prediction of a candidate interaction between M and H, *if* information about the details of the interaction (i.e. the specific regions involved in the interaction interface) is not taken into account. Given that resolved crystal structures are available for only a small fraction of the PPIs known to date [37], this somewhat “blind” approach could lead to false inferences, considering that the human, and even MTB, proteome includes a significant proportion of multi-domain proteins (Additional file 1: Figure S1).

On the other hand, imposing stringent filters on the coverage of the template sequence in the alignment in order to avoid the above caveat might turn out to be too restrictive. This constraint could cause potential interactions to be missed out, if the query and template proteins happen to share a highly conserved region that is sufficient for the template interaction to occur.

Such pitfalls underscore the need to incorporate additional information about the structural interface actually mediating the template interaction into sequence-based search for viable PPI candidates, which can improve the quality of the predictions. Assuming that functional domains are independently folding modular units of protein structure and mediate inter-protein interactions, restricting the template PPIs to the subset for which structural information is available provides a more targeted approach to search for potential PPIs. We propose leveraging the structurally resolved protein complexes deposited in the Protein Data Bank (PDB), and using their interacting portions (domains) instead of the full length protein sequences as templates in a local alignment-based search for viable human-MTB PPIs. Combined with other sources of information such as functional annotation, cellular localization and cell type-specific gene expression data [38], such an approach has the potential to suggest novel, high-confidence candidates for in vivo interactions, which could contribute to filling the gaps in our understanding of the disease process.

## Methods

### Preparation of host and pathogen protein sets

The proposed method screens for physical protein-protein interactions between human and MTB proteomes. Complete reference proteomes for *H. sapiens* and the virulent *M. tuberculosis* strain H37Rv along with functional meta-data for the proteins were downloaded from UniProt (Sept 2015). All human proteins were retained for the subsequent search. On the other hand, the MTB proteome was restricted to a smaller subset composed of proteins for which there is direct or indirect link to infection/adaptation inside the host (and which thus are plausible candidates for physical interactions with human proteins). In order to construct this subset, we merged the following sources of contextual information for MTB: (1) proteins annotated with a select set of relevant keywords or GO cellular component terms in UniProt (*extracellular, secreted, cell wall, cell surface, antigen, host, macrophage, monocyte*); (2) all proteins detected in the culture filtrate in the proteomic study by de Souza et al. [39]; (3) all proteins containing an export signal sequence as predicted by the PSORTb (v3.0) tool [40]; and (4) all MTB ORFs which had been pre-selected on the basis of literature curation for the Y2H screening experiment in Mehra et al. [21]. The union of these datasets provides a total of 1059 MTB proteins, covering nearly 25% of its proteome.

### Structural information about domain-domain interactions

Information about domain-domain interactions in PDB protein complexes was obtained from the iPfam (version 1.0) and 3DID (version 2015_02) databases [41, 42]. Both these resources infer the presence of interactions between Pfam-A domains [43] within and across subunits based on residue-residue distances (and biochemical compatibility) in the corresponding resolved three-dimensional structures. For increased stringency, we only retained the inter-chain DDI information, and this list was futher pruned to only include those domain pairs which were present in (and thus could mediate interactions between) the pre-selected MTB protein set and the human proteome. Polypeptide sequences of these domains as well as the UniProt accession numbers of their parent proteins were extracted from the corresponding PDB files.

### Comparison of MTB proteins with their orthologs

In order to highlight the potential limitations of local sequence alignment for PPI inference, we identified Reciprocal Best Hits (RBH) for every protein in the MTB set using NCBI protein BLAST search (run with default parameters, and a stringent E-value threshold of 1e-10) against the SwissProt database. RBH provides the best match for a query protein in every other annotated proteome, and this approach is routinely employed in comparative genomics to screen for orthologs [44–46]. Statistics for the RBH hits (pair-wise sequence similarity and percentage coverage in the alignment) were obtained from the BLASTP output file. Additionally, similarity on the domain level between every protein and its RBH partner was quantified in terms of the jaccard index for the overlap between their Pfam domain sets, which is a number between 0 (no common domain) and 1 (identical domain composition). Besides the RBH approach, we separately obtained the above statistics for the predicted orthologs of MTB proteins retrieved from three other databases: Integr8 [47], eggNOG [48] and KEGG Orthology [49].

### Sequence-based approach to prioritization of candidate host-pathogen PPIs

We used Smith-Waterman (SW) local alignment implemented in the EMBOSS command-line tool [50] (BLOSUM62 substitution matrix, gap open penalty = 10, gap extension penalty = 0.5) to scan the MTB/human proteins for close matches with the interacting template sequences. If a subsequence in an MTB protein (b) had x% similarity with a template domain sequence T, and a human protein (h) contained a subsequence y% similar to the interacting partner of T, then the joint score S for the pair (b, h) was calculated as the geometric mean of x and y, i.e. S = √xy. Our choice of this measure follows from [28], although we use similarity rather than the more restrictive sequence identity, since substitution of residues by other physicochemically similar residues should still provide a good guide for structure-level closeness. In addition, we imposed the somewhat stringent, uniform constraint that at least 90% of each template sequence be covered by the respective alignment. Pathogen-host protein pairs were ranked according to their scores. A pair could get multiple scores in principle, but every score was treated as independent; thus, only the best score for every pair finally matters. High-scoring pairs are assumed to represent viable candidates for physical protein-protein interaction, which could be probed further through follow-up studies for their possible relevance to the infection process.

### Integrating large scale PPI data with evolutionary information to enlarge the search space

Since our template dataset only includes PPIs with 3D structural information, it is quite restricted in size. We considered extending the coverage of our approach by assuming that evolutionarily conserved interactions across different species share a common underlying pattern of domain-domain interactions. Thus, we looked for such conserved interactions among the much larger collection of PPIs for which some form of experimental evidence (but not necessarily resolved structure) is available. This procedure is outlined in Additional file 1: Figure S3. If a functional PPI has been reported to occur between proteins A’ and B’, where A’ and B’ are orthologs of an interacting pair (A, B) that is part of a structurally resolved complex, then it is assumed that the interaction between A’ and B’ is mediated by the same DDIs as those between (A, B), and these domain sequences were added to the template set. Literature evidence for PPIs was obtained from the following online resources: IntAct, MINT, BioGrid, DIP, HPRD and HPIDB [51–56]. The merged (non-redundant) dataset comprised a total of ~ 400,000 PPIs involving ~ 66,700 proteins, and the interolog search, based on the InParanoid database [57], yielded about 1830 additional domain sequences for use as templates. Although this exercise does not contribute novel domain-domain interactions that are not already present in the original template set, the inclusion of more template sequences further improves the chances of finding high-scoring candidates with the alignment-based search.

**Fig. 3:**
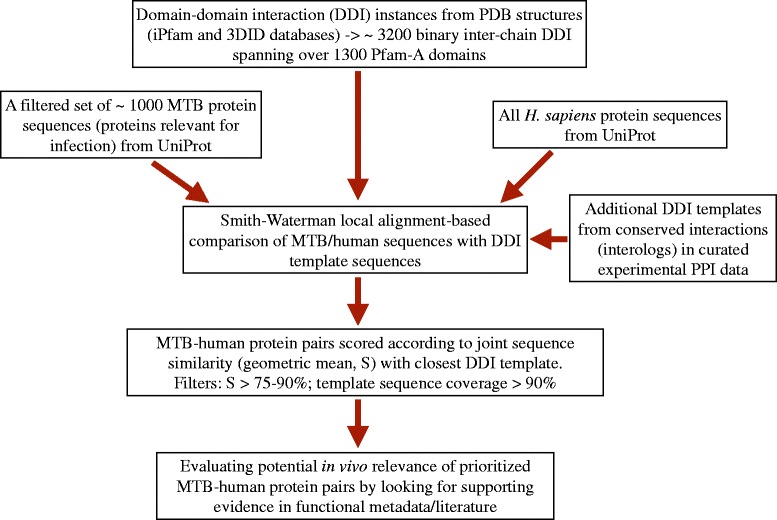
Summary of the steps involved in our sequence/domain-based approach to the search for potentially novel (uncharacterized) human-microbial PPI

The various steps involved in our methodology have been summarized as a flow chart in Fig. 3.

### Quantifying interaction specificity on the domain level, and diversity within domain families

Domain-domain interactions can be specific, and thus, it is quite likely that every instance of a particular pair of domains, for which interaction evidence is found in a few PDB complexes, will not engage in a detectable interaction [58]. We present the following data to highlight these differences within Pfam-A domain families:

1. We randomly selected 50 domains that each occur 30–60 times among the proteins comprising the large scale PPI dataset (see above). Differences among the members of every domain family were quantified in terms of their pair-wise sequence similarity values (based on SW alignment), and the pair-wise differences in sequence length scaled by the average length for that family. The distributions of these metrics are illustrated for the particular example of the Ulp1 protease family C-terminal catalytic domain (PF02902) in Additional file 1: Figure S4.
2. We estimated the statistical association between the interacting domains (as well as domain combinations) listed in iPfam/3DID and a large scale physical PPI network for *E. coli.* Briefly, the frequency of occurrence of every domain pair in the positive set (i.e. among the PPIs) was compared with its incidence in the complement set (that comprised all possible pairs of proteins for which no interaction evidence has been reported). Statistical significance of its over-representation in the PPI set was assessed in terms of the p-value estimated by one-sided Fisher’s exact test. There could be protein pairs for which no interaction evidence has been reported because they are present in different cellular locations or not co-expressed under in vivo conditions, but nevertheless could still be compatible to physically interact in vitro. Thus, not all members of the complement set are expected to be bona fide non-interactions. In order to minimize the influence of this confounding factor on estimation of likelihood ratios, and refine the complement set to better reflect the “true” negative set, we retained only those *E. coli* protein pairs in the complement set which were co-localized to the cytosol (as indicated by their GO cellular component annotation), and showed correlated expression (pearson correlation coefficient > 0.5) in the M3D compendium of microarray profiles [59].

## Results

### Limitation of local sequence alignment for knowledge-based PPI inference

**Fig. 4:**
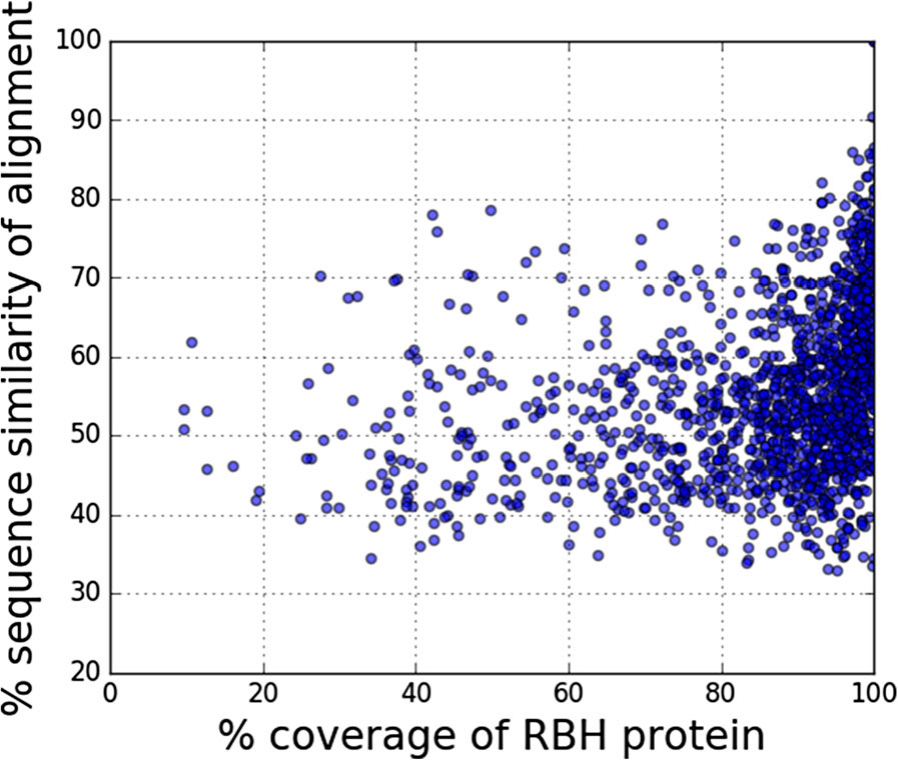
Pair-wise comparison between MTB proteins and their Reciprocal Best Hits (RBH) in the SwissProt database identified by two-way pBLAST search. In a number of cases, the RBH protein sequence is only partially covered by the corresponding local alignment

Local alignment of two sequences looks for matching subsequences in longer full length proteins. Consequently, even matches which cover only a fraction of the template protein sequence can score high in terms of the statistical significance (E-value) or percentage sequence similarity of the aligned portion. If the template PPI set contains a substantial proportion of proteins having more than one functional domain, the possibility of ascribing spurious interactions to protein pairs exists in the absence of structural details about the template interaction (Fig. 2). In order to illustrate this, we have identified the Reciprocal Best Hits (RBH) across a large number of genomes for a filtered set of MTB proteins which might have some role to play in the infection process (Methods). Pairwise comparison between RBH proteins has been quantified in terms of the sequence similarity of the local alignment and the percentage of the hit sequence covered by the alignment. This is displayed as a two-dimensional scatter plot in Fig. 4. The considerable scatter along the horizontal axis, even for hits with highly significant E-values, suggests that partial coverage of the hit protein sequence (which could be a participant in a template interaction) leaves room for ending up with off-target matches as in the example in Fig. 2. Particularly when comparing bacteria with eukaryotes, one is likely to come across instances of evolutionarily related proteins having diverged, and functionally diversified, considerably. We further note that the pair-wise similarity values also have a broad distribution, a point we shall elaborate on in what follows.

**Fig. 5:**
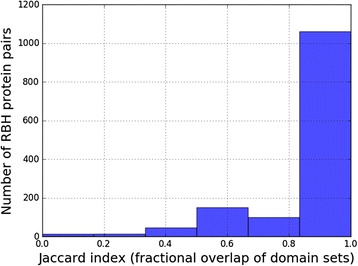
Similarity on the level of domain composition between RBH protein pairs. The jaccard index is a number between 0 (no shared domain) and 1 (identical domain sets)

Another way to depict the differences between proteins related by RBH is shown in Fig. 5, which summarizes pair-wise comparisons between proteins in terms of their domain composition. The overlap between domain sets is quantified by the jaccard index, which is maximal (=1) when they are identical. Nearly a quarter (24%) of the protein pairs identified by RBH do not share identical domain composition, once again underscoring the need to factor in structural (DDI) information when proposing potential interactions based on template PPIs. Similar results are also found with other resources for orthologous proteins, such as Integr8 (which was the basis for an earlier publication on human-MTB PPI prediction [20]), eggNOG and KEGG Orthology (Additional file 1: Figure S2).

### Using structural information in a targeted search for candidate host-MTB protein interactions

Knowledge about interacting domains extracted from functional complexes with structural information can be used to suggest potential interactions between proteins for which experimental interaction evidence is lacking. We have made use of the set of structurally resolved protein complexes listed in two databases, *i*Pfam and 3DiD [41, 42], as our background dataset for domain-level interaction information. This collection is comprised of a diverse set of interaction instances, including homo and hetero-domain, intra and inter-subunit, and multi-protein as well as dimeric interactions. About 77% of the inter-subunit interactions are mediated by just a single pair of domains. At the other extreme, as many as 16 pairs of interacting domains between the same two subunits (PDB chains) have been inferred from inter-residue distances in some of the complexes (Additional files 2 and 3).

We have restricted our search for potential human-MTB PPIs to a filtered set of ~ 1000 MTB proteins which may have some functional role to play in the infection process (Methods). *i*Pfam and 3DiD taken together provide a list of 3265 binary domain-domain (inter-chain) interactions involving 1356 Pfam domains. Out of these, 1034 domains have at least one instance of occurrence in the human/filtered MTB proteomes, and they collectively yield a total of ~ 82470 pairs of MTB and human proteins that could potentially interact.

Despite a reduction by nearly 3 orders of magnitude, this still represents a fairly large number of possibilities. Moreover, no distinction is being made between the different instances of a *single* domain pair for which interaction evidence has been found in only a few (or just one) PDB structures. Therefore, we suggest a simple scoring scheme to sort these plausible interactions, using the evidence from the known DDI interfaces as a prior. The sequences corresponding to the interacting DDI instances have been extracted from the corresponding PDB files and used as templates to scan the human and MTB proteomes. Each “hit” has been scored in terms of the coverage of the template sequence in the resulting alignment (required to be > 90%) and sequence similarity with the template in the alignment. For different thresholds of varying stringency, the search yields a small set (10–500) of prioritized MTB-host protein pairs which could represent functional PPIs (Table 1).

**Table 1:** Summary of results of the sequence-based search for candidate PPIs between MTB and human, for different score thresholds

Joint similarity score threshold	Number of candidate pairs	Number of MTB proteins	Number of human proteins
0.75	464	103	313
0.8	178	50	141
0.9	11	5	9

The template set, which is composed of DDI instances extracted from PDB structures, represents only a small fraction of the known, empirically validated PPIs reported in the literature. This imposes a limitation on our approach, since MTB-human protein pairs are being scored by how “close” they are on the sequence level to template DDI instances. In order to increase the number of template DDI sequences available to search against and thereby increase the chances of finding high scoring MTB-host protein pairs, we suggest leveraging the much more extensive data available on experimentally known PPIs, by looking for conserved interactions (“interologs”) across species. The pattern of domain-domain interactions mediating the PPI is also assumed to be conserved, and this provides additional template sequences to compare against (Additional file 1: Figure S3). Based on a collection of ~ 400,000 experimentally validated binary physical protein-protein interactions which integrates multiple PPI databases (see Methods), an additional ~ 1830 template domain sequences were obtained, and this set contributes a further 23 MTB-human protein pairs with scores > 0.8 (Table 2).

**Table 2:** Prioritized candidate MTB-human PPIs suggested by the template domain-domain interactions that were derived from the interologs of the structurally resolved complexes in iPfam/3DID

Joint similarity score threshold	Number of candidate pairs (number unique to interolog-based search)	Number of MTB proteins	Number of human proteins
0.75	168 (30)	64	120
0.8	87 (23)	29	73
0.9	4 (0)	2	3

### Diversity within domain families and domain-level interaction specificity

**Fig. 6:**
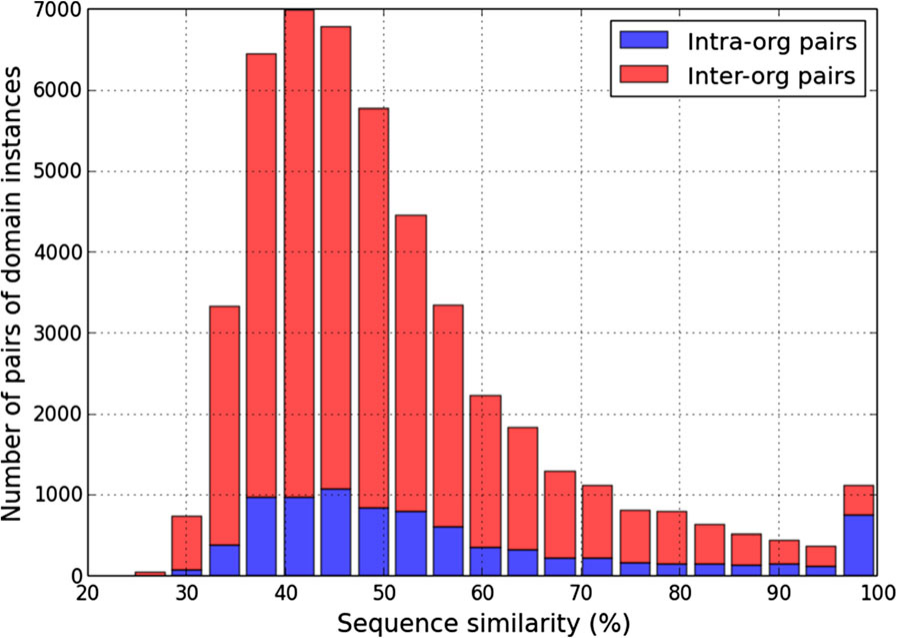
Diversity on the sequence level within domain families. Histogram of the percentage sequence similarity values resulting from all-vs-all pairwise alignment of members assigned to a common domain. The distribution represents the aggregate of a random selection of 50 Pfam domains with 30–60 members in each family

Our approach to the identification of potentially interacting sequence pairs is based on a local search in sequence space in the neighborhood of known interacting domain instances. For a given pair of Pfam domains for which interaction evidence exists in the PDB, not all instances of the same two domains may interact equally effectively to be of functional consequence [58]. Variation in the detailed structure across different amino acid sequences assigned to a common functional domain may be expected to give a broad distribution in terms of interaction strength across the different instances. Empirical support for such interaction specificity is suggested by the following observations:

- Sequences identified with a common Pfam domain can show considerable diversity in terms of both length and sequence composition. This is suggested by the histogram in Fig. 6, which represents the aggregate of pair-wise sequence similarity values estimated within ~ 50 Pfam domain families with at least 30 instances (distinct sequences) in each family. Two sequences assigned to the same domain can differ by as much as 70%, and there is no discernible difference between sequence-level conservation within and across species (red versus blue distribution). Our approach attempts to tap the high similarity end of this distribution to find domain sequences in the MTB/human proteomes that closely match the known interactors.
- Some domains (and even domain combinations) showing interaction evidence in PDB complexes also occur in non-interacting protein pairs listed in the Negatome database [60]. This curated collection comprises ~ 2000 pairs of mammalian proteins for which lack of interaction was reported in small scale experimental studies. The Negatome is a potential source of negative training data for use in supervised interaction prediction algorithms.
- Several domain pairs inferred to interact from structural information in specific instances, do not show statistically significantly association (adjusted *p*-value > 0.05) with the set of known PPIs among the co-localized, co-expressed proteins in *E. coli* (see Methods). Such a lack of statistical enrichment is found not only for domain pairs but also for some pairs of domain combinations (Additional file 1: Figure S5). Although it could well be the result of incomplete coverage and noise in the PPI dataset used, this observation is also consistent with the idea of interaction specificity arising from finer structural differences among the members of a domain family [58].

The above observations, taken together, suggest differences in the ability to effectively interact across different instances of the same pair of domains (or domain combinations). Our scoring scheme based on sequence similarity provides a simple way to factor in this heterogeneity, and sort the list of host-pathogen protein pairs according to the empirical evidence for domain-domain interactions currently available in the PDB.

### Functional characterization of the prioritized host-MTB PPI candidates

**Fig. 7:**
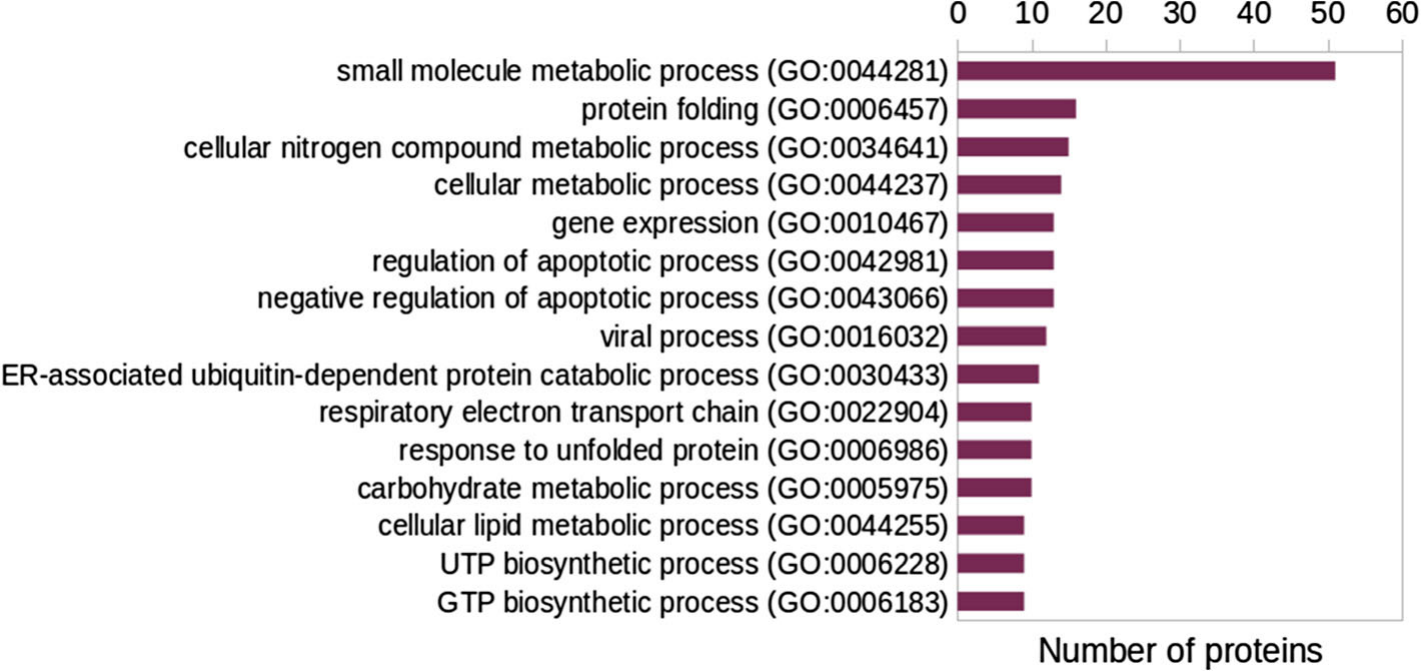
Functional annotation of top-ranking human proteins. The host proteins appearing in the prioritized list of PPI candidates (score threshold = 0.8) were assigned GO annotation. The 15 most frequently occurring GO biological process terms are shown here

**Fig. 8:**
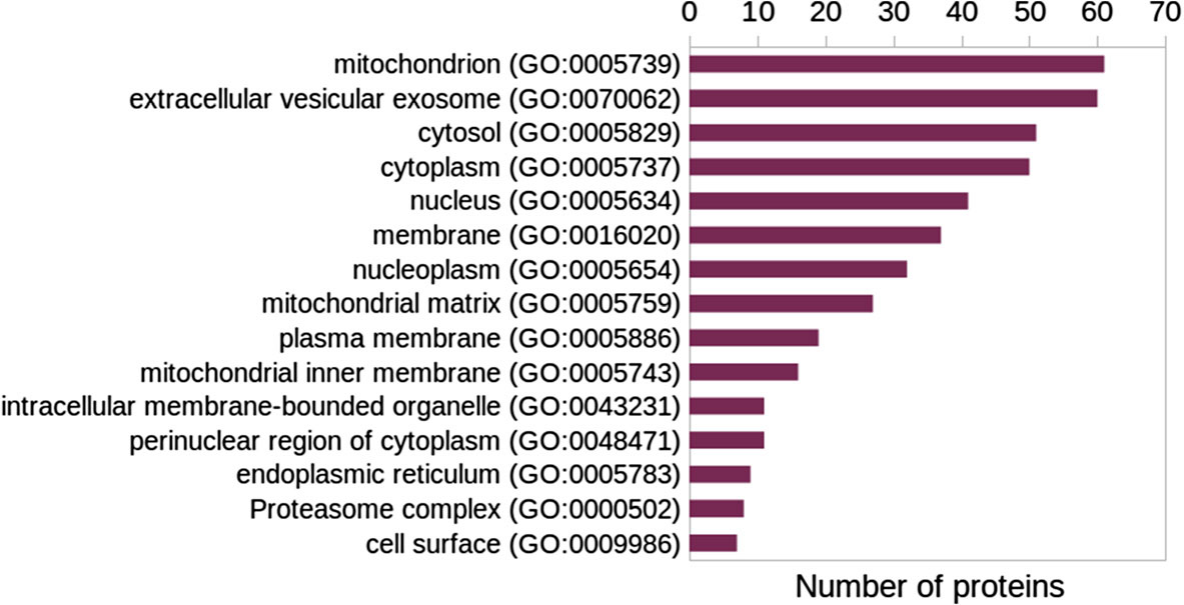
Cellular distribution of top-ranking human proteins. The host proteins appearing in the prioritized list of PPI candidates (score threshold = 0.8) were assigned GO annotation. The 15 most frequently occurring GO cellular component terms are shown here

We have arrived at a non-redundant, prioritized list of MTB-human protein pairs, each of which contains at least one pair of subsequences closely resembling a known DDI instance on the sequence level. The complete list of predictions may be found in Additional files 4 and 5, and the top-ranked protein pairs (score threshold of 0.9) are listed in Table 3. (We attempted a comparison of our predictions with those from previously published (most recent) studies, the outcome of which is summarized in Additional file 6 for the reader’s convenience.) These pairs represent candidates for physiologically relevant protein-protein interactions. The template DDIs are composed of both homo and hetero domain interactions, but were restricted to only include inter-chain interfaces. The sorted list contains a substantial fraction of interactions involving heat shock proteins/chaperones, redox proteins such as dehydrogenases, and nucleoside diphosphate kinases. In order to gain a better idea about the overall functional landscape of the filtered host proteins and possible roles in processes relevant to infection, we have retrieved their Gene Ontology biological process (BP) and cellular component (CC) annotations. The most frequently appearing GO BP and CC terms are represented in the bar plots in Figs. 7 and 8. Pathways associated with known perturbations of the host macrophage following infection [7], such as regulation/negative regulation of apoptotic process (GO:0042981/GO:0043066), cellular lipid metabolic process (GO:0044255), ER-associated stress response (GO:0030433) and respiratory electron transport chain (GO:0022904), occur several times among the identified host proteins. Another possible determinant of the in vivo relevance of predicted interactions is the cellular location of the involved host proteins. A number of candidate interactors present in our list are associated with cellular components known to be relevant for infection [13, 14], such as mitochondrion (GO:0005739)/mitochondrial matrix (GO:0005759), endoplasmic reticulum (GO:0005783) and cell surface (GO:0009986).

**Table 3:** Top-ranked pairs of MTB and Human proteins identified by our approach (filtered at similarity score threshold of 0.9 and ordered by score)

MTB UniProt Acc.	Human UniProt Acc.	MTB Protein symbol(s)	Human protein symbol(s)
P9WPU5	P25705	atpD/Rv1310/MTCY373.30	ATP5A1/ATP5A/ATP5AL2/ATPM
P9WPU5	P36542	atpD/Rv1310/MTCY373.30	ATP5C1/ATP5C/ATP5CL1
P9WNN1	P49411	tuf/Rv0685/MTCY210.02	TUFM
P9WPU7	P25705	atpA/Rv1308/MTCY373.28	ATP5A1/ATP5A/ATP5AL2/ATPM
P9WPU7	P06576	atpA/Rv1308/MTCY373.28	ATP5B/ATPMB/ATPSB
P9WPU7	P36542	atpA/Rv1308/MTCY373.28	ATP5C1/ATP5C/ATP5CL1
P9WNG9	Q5T4U5	etfA/fixB/Rv3028c/MTV012.43c	ACADM/hCG_22915
P9WNG9	P38117	etfA/fixB/Rv3028c/MTV012.43c	ETFB/FP585
P9WNG9	B7Z9I1	etfA/fixB/Rv3028c/MTV012.43c	ACADM
P9WNG9	P11310	etfA/fixB/Rv3028c/MTV012.43c	ACADM
P9WMJ9	P38646	dnaK/Rv0350/MTCY13E10.10	HSPA9/GRP75/HSPA9B/mt-HSP70

We further sought circumstantial evidence for the in vivo relevance of the prioritized interactions. To this end, we prepared a list of 462 relevant human proteins by combining datasets on host dependency factors [61], host cell signaling pathways engaged during infection (KEGG) [49], and proteins coded by the human genes that are linked with susceptibility to TB (OMIM) [62]. These proteins collectively comprise a functional subnetwork that determines the host response to infection. We find 4 of these proteins involved in 8 plausible interactions with MTB proteins (scores > 0.7). These include HSPA9 (Stress-70 protein, mitochondrial) and HSPD1 (60 kDa Mitochondrial heat shock protein) which occur in the KEGG Tuberculosis pathway [49], and Vcp (Transitional ER ATPase) and ATP5C1 (ATP synthase subunit gamma, mitochondrial) which have been previously identified as being essential for survival of MTB inside THP-1 cells [61]. However, these putative interactions are mediated by homo-domain DDIs, and merely involve a human protein displaced by a closely related (functionally homologous) MTB protein in a multi-protein complex (as an example, the heat shock protein HSPA9 is predicted to be targeted by Dnak/Rv0350, one of the heat shock proteins in MTB). Thus, they are unlikely to lead to non-trivial physiological alterations in the host cell.

Our approach picks out a few potential interactions which could be of some interest. The secreted Hypoxia response protein 1 of MTB (Rv2626c) is predicted to interact with CLCN3 (H(+)/Cl(−) exchange transporter 3 (Chloride channel protein 3)) through their CBS domains (PF00571). This latter protein is an antiporter associated with the endosomal/phagosomal membrane, and contributes to the acidification of the endosomal lumen thereby possibly affecting vesicle trafficking [63]. GO annotation also suggests a role for it in regulation of ROS biosynthesis (GO:1903428, positive regulation of reactive oxygen species biosynthetic process). Thus this putative interaction may be of relevance to the survival of virulent MTB inside the phagosome and alteration of phagosomal maturation. Another candidate interaction involves a cell wall-associated putative conserved ATPase (Rv0435c) in MTB and the human vacuolar protein sorting-associated protein 4B, VPS4B, mediated by the Pfam domain pair AAA/ATPase family associated with various cellular activities (PF00004) and Vps4_C/Vps4 C terminal oligomerisation domain (PF09336) (alternatively, also by a homo-domain DDI between the AAA domains of the two proteins). VPS4B is involved in the endosomal multivesicular body (MVB) sorting pathway that regulates endosome to lysosome transport, and it has been previously found to have a role in enveloped viral budding (HIV-1 and other lentiviruses) from the host cell [64]. We note that another related vacuolar sorting-associated protein, VPS33B, happens to be a known substrate for the secreted MTB phosphatase PtpA. This interaction was earlier shown to inactivate VPS33B leading to inhibition of acidification of the mycobacterial phagosome [65]. The plausible interaction with VPS4B found here also may have a role to play in altered vesicular trafficking following phagocytic engulfment of MTB.

## Discussion

Genome-based computational methods have been employed for several years now to help reconstruct the protein interactome underlying the functional landscape in a number of organisms [25–27, 30]. More recently, attention has been turned to the problem of predicting PPIs between pathogenic microbes and the human host towards gaining a better understanding of infectious diseases [23, 24]. Comparative methods based on local sequence alignment [31–34] are bound to yield a significant proportion of false positives, or negatives, unless structural (domain-level) details about the template interactions are also properly taken into account. However, structural information is currently available for only a small proportion of the experimentally validated PPIs [37]. This restricts the size of the template dataset to work with, and is the price to be paid for improved specificity of local alignment-based search.

We have demonstrated how the structurally resolved complexes in the PDB [41, 42] can be tapped to suggest potential interactions between host and pathogen proteins, and applied it to the specific case of *M. tuberculosis*. Our targeted approach may be viewed as setting an upper bound on the performance of any comparative sequence-based method for identifying PPI candidates that relies purely on sequence alignment. We have proposed reducing the occurrence of false positives (esp. in the case of multi-domain proteins) and negatives, and increasing specificity, by prioritizing the candidate interactions on the basis of their domain-level sequence similarity with the template proteins. On the other hand, since our approach is extrapolatory in nature, only those candidate protein pairs which share similar subsequences with the template interactors get high scores. Thus, the number of high-scoring predictions that can be made, i.e. the sensitivity of the method, is limited by the size and diversity of the template dataset used. (We note, for instance, that our prioritized list does not include any of the ~ 40 PPIs between MTB and human that are experimentally known so far [20].) However, as the number of resolved crystal structures deposited in the PDB continues to grow at an ever increasing pace, we expect the scope and utility of our methodology to also improve with time, and it has the potential to provide an efficient and cost-effective alternative to experimental high-throughput screens for interactome mapping [21, 22].

Some earlier studies have also proposed the use of domain-level interaction information to screen for potential PPIs, but those approaches essentially treat all members of a domain family on the same footing, disregarding the differences in interaction ability among them [32, 33, 66–69]. Thus, all occurrences of a pair of domains that could potentially interact are weighted equally. However, not every instance of a pair of domains is likely to engage in a functional interaction [58]. This anticipated heterogeneity provides the rationale for our scoring scheme to sort the candidate protein pairs. Determining whether a functional interaction can occur between a pair of protein sequences is, of course, a difficult question, and requires extensive biochemical characterization which is beyond the scope of the present study. We have adopted a pragmatic approach and searched for proteins in the human/MTB proteomes which are “close enough” in sequence space to known functional interactions retrieved from the PDB. In a sense, the score can be regarded as a proxy for the likelihood that an interaction will occur between the two candidate proteins, although we emphasize again that low score does not by itself imply non-interaction - improved specificity comes at the cost of limited sensitivity.

**Fig. 9:**
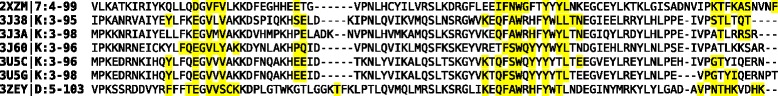
Interfacial residues mediating a common domain-domain interaction in different complexes do not necessarily line up in the multiple sequence alignment. The displayed alignment corresponds to the Pfam domain Plectin/S10 (PF03501), which is found to interact with the K Homology domain (PF07650) in several PDB complexes. The sequences (domain instances) are labeled by their PDB identifiers (PDB ID, chain ID and residue numbers). The interfacial residues involved in this DDI are highlighted in yellow, and their positions in the alignment show considerable scatter

The candidate interactions could have been prioritized in other ways, besides the one we have adopted. For example, the joint similarity metric could be calculated based on only those positions in the sequence alignment that correspond to the directly interacting residues in the template complex (as was done in [70], for example). This of course implicitly assumes that the interfacial residues mediating a domain-domain interaction in different protein structures all line up when the corresponding sequences are aligned, and thus, only these residues would be relevant for making comparisons. We tested this assumption on examples of interacting domains which have multiple instances of occurrence in iPfam/3DID. Sequence alignment of the corresponding domain sequences suggests that the interacting residues do not always align, and there can be considerable scatter. This is illustrated for the particular case of the Plectin/S10 domain (PF03501) in Fig. 9. The interfacial residues (extracted from different PDB structures) mediating its interaction with the KH domain (PF07650) are highlighted in yellow in the multiple sequence alignment, and it is evident that restricting the comparison to only those positions which align with the interacting residues in the template, is likely to result in fairly meaningless inferences. We have based our comparisons on the full domain sequences, because the overall three-dimensional structure of the domain and spatial arrangement of its residues – which decides the nature of the active site and energetics of inter-protein interactions - is after all an emergent ‘collective’ property of the polypeptide chain, arising from the overall physico-chemical composition of its entire sequence.

Finally, we note that the potential relevance of the high-scoring leads picked out by our sequence-based search could be further assessed by integration with other sources of contextual information besides Gene Ontology, such as large scale gene expression changes and knowledge about the host interactome. For example, several methods have been recently developed to infer the causal upstream regulators (e.g. DNA-binding transcription factors) that might underlie changes in the transcriptional profile at various stages of the infection [71–73]. With the aid of curated large scale signaling networks [74], it might be possible to discover novel links between such alterations in regulatory activity and some of the computationally predicted host targets of pathogen proteins. Such an integrative analysis, which will be reported elsewhere, could suggest novel hypotheses regarding the molecular pathways that shape the temporal course and eventual outcome of the disease.

## Conclusions

Our analysis of local sequence alignment applied to host-pathogen PPI prediction highlights the possibility of drawing spurious inferences (or missing out on potential interactions), if structural details about the template interactions are not available/not taken into account. We have proposed making use of the structurally resolved complexes in the Protein Data Bank for more targeted search for novel PPI candidates between human and MTB proteins. The use of domain-domain interaction information reduces the chances of false positives/negatives from local sequence alignment-based PPI prediction. Our knowledge-based approach, which looks for similar sequences in the vicinity of known DDI templates, acknowledges the inherent diversity within domain families and DDI interaction specificity, for which we have provided different lines of supporting data. Although we have illustrated our methodology with the specific case study of *M. tuberculosis,* it is of general applicability, and should provide a useful data-driven approach to predicting and prioritizing potential PPIs between any pathogenic microbe and its host that leverages the existing genomic and structural datasets available in the public domain.

## Additional files

Additional file 1: Figure S1. Histograms summarizing the occurrences of Pfam-A functional domains in the human (blue) and MTB (red) proteomes. Nearly 30% of the proteins in both species have been assigned more than one domain. **Figure S2.** Pair-wise comparisons between MTB proteins and their putative orthologs listed in the Integr8 (upper figure), eggNOG (lower left) and KEGG Orthology/KO (lower right) databases. Note the relatively smaller number of orthologs assigned by Integr8. **Figure S3.** Using the interolog approach to extend the limited set of DDI templates provided by PDB. Evolutionarily conserved PPIs (involving orthologs) are assumed to share a common underlying pattern of domain-domain interactions. This allows interacting domains to be inferred for PPIs for which structure information is not directly available (here, A’-B’). **Figure S4.** Diversity in terms of length and sequence composition across the polypeptide sequences assigned to the Ulp1 protease family C-terminal catalytic domain (PF02902). The scatter plot follows from all-vs-all pairwise sequence alignment using the Smith-Waterman method. Pairs coming from the same proteome (blue) or from different proteomes (cyan) have been assigned different colors. **Figure S5.** Statistical over-representation of interacting Pfam domain pairs (from iPfam/3DID) in a PPI network for co-localized, coexpressed cytosolic proteins in *E. coli.* Several domain pairs do not show significant association with the PPI set. The darker horizontal line represents the *p*-value < 0.05 threshold. (PDF 1100 kb)

Additional file 2: Complete list of domain-domain interactions from *i*Pfam. Column headers: 1) Pfam ID of domain 1; 2) Pfam ID of domain 2; 3) PDB chain ID of domain 1; 4) PDB chain ID of domain 2; 5) Uniprot accession of PDB chain 1; 6) Uniprot accession of PDB chain 2. (TXT 6425 kb)

Additional file 3: Complete list of domain-domain interactions from 3DID. Column headers: 1) Pfam ID of domain 1; 2) Pfam ID of domain 2; 3) PDB chain ID of domain 1; 4) PDB chain ID of domain 2; 5) Uniprot accession of PDB chain 1; 6) Uniprot accession of PDB chain 2. (TXT 6075 kb)

Additional file 4: Prioritized list of human-MTB protein pairs obtained by comparison with PDB DDI templates, sorted according to their joint similarity scores (>0.7 only). Colum headers: 1: Uniprot accession of MTB protein. 2: Uniprot accession of human protein. 3: Alternate symbols for MTB protein. 4: Alternate symbols for human protein. 5: Template sequence (PDB chain ID) mapping to MTB protein. 6: Template sequence (PDB chain ID) mapping to human protein. 7: Pfam ID of template mapping to MTB protein. 8. Pfam ID of template mapping to human protein. 9: Pfam domain name of template mapping to MTB protein. 10. Pfam domain name of template mapping to human protein. 11. Uniprot accession of template mapping to MTB protein. 12: Uniprot accession of template mapping to human protein. 13: Joint similarity score from comparison with the template DDI. 14: Total number of DDIs between the two interacting proteins to which the template DDI belongs. (TXT 7871 kb)

Additional file 5: Prioritized list of human-MTB protein pairs obtained by comparison with the interlog-derived template set, and sorted according to their joint similarity scores (>0.7 only). Colum headers: 1: Uniprot accession of MTB protein. 2: Uniprot accession of human protein. 3: Alternate symbols for MTB protein. 4: Alternate symbols for human protein. 5: Template sequence (SwissProt ID|Pfam ID|sequence) mapping to MTB protein. 6: Template sequence (SwissProt ID|Pfam ID|sequence) mapping to human protein. 7: Pfam domain name of template mapping to MTB protein. 8. Pfam domain name of template mapping to human protein. 9: Joint similarity score from comparison with the template DDI. 10: Total number of DDIs between the two interacting proteins to which the template DDI belongs. (TXT 204 kb)

Additional file 6: Summary of comparison of our predictions with those from four most recently published computational studies on host-MTB interactions. (PDF 49 kb)

## Abbreviations

DDI: Domain-domain interaction
MTB: *Mycobacterium tuberculosis*
PPI: Protein-protein interaction
RBH: Reciprocal best hit
