# Supplementary material for: Using structural knowledge in the protein data bank to inform the search for potential host-microbe protein interactions in sequence space: application to Mycobacterium tuberculosis
Source: BMC Bioinformatics. 2017 Apr 4;18:201. doi: 10.1186/s12859-017-1550-y (PMC5379762; doi:10.1186/s12859-017-1550-y)
Supplement: Supplementary file 1 — Histograms summarizing the occurrences of Pfam-A functional domains in the human (blue) and MTB (red) proteomes. Nearly 30% of the proteins in both species have been assigned more than one domain. Figure S2. Pair-wise comparisons between MTB proteins and their putative orthologs listed in the Integr8 (upper figure), eggNOG (lower left) and KEGG Orthology/KO (lower right) databases. Note the relatively smaller number of orthologs assigned by Integr8. Figure S3. Using the interolog approach to extend the limited set of DDI templates provided by PDB. Evolutionarily conserved PPIs (involving orthologs) are assumed to share a common underlying pattern of domain-domain interactions. This allows interacting domains to be inferred for PPIs for which structure information is not directly available (here, A’-B’). Figure S4. Diversity in terms of length and sequence composition across the polypeptide sequences assigned to the Ulp1 protease family C-terminal catalytic domain (PF02902). The scatter plot follows from all-vs-all pairwise sequence alignment using the Smith-Waterman method. Pairs coming from the same proteome (blue) or from different proteomes (cyan) have been assigned different colors. Figure S5. Statistical over-representation of interacting Pfam domain pairs (from iPfam/3DID) in a PPI network for co-localized, coexpressed cytosolic proteins in E. coli. Several domain pairs do not show significant association with the PPI set. The darker horizontal line represents the p-value < 0.05 threshold. (PDF 1100 kb) [file 12859_2017_1550_MOESM1_ESM.pdf]

List of supplementary figures:

Figure S1:
Histograms summarizing the occurrences of Pfam-A functional domains in the human (blue) and MTB (red) proteomes. Nearly 30 % of the proteins in both species have been assigned more than one domain.

Figure S2:
Pair-wise comparisons between MTB proteins and their putative orthologs listed in the Integr8 (upper figure), eggNOG (lower left) and KEGG Orthology/KO (lower right) databases. Note the relatively smaller number of orthologs assigned by Integr8.

Figure S3:
Using the interolog approach to extend the limited set of DDI templates provided by PDB. Evolutionarily conserved PPIs (involving orthologs) are assumed to share a common underlying pattern of domain-domain interactions. This allows interacting domains to be inferred for PPIs for which structure information is not directly available (here, A'-B').

Figure S4:
Diversity in terms of length and sequence composition across the polypeptide sequences assigned to the Ulp1 protease family C-terminal catalytic domain (PF02902). The scatter plot follows from all-vs-all pairwise sequence alignment using the Smith-Waterman method. Pairs coming from the same proteome (blue) or from different proteomes (cyan) have been assigned different colors.

Figure S5:
Statistical over-representation of interacting Pfam domain pairs (from iPfam/3DID) in a PPI network for co-localized, coexpressed cytosolic proteins in *E. coli.* Several domain pairs do not show significant association with the PPI set. The darker horizontal line represents the p-value < 0.05 threshold.

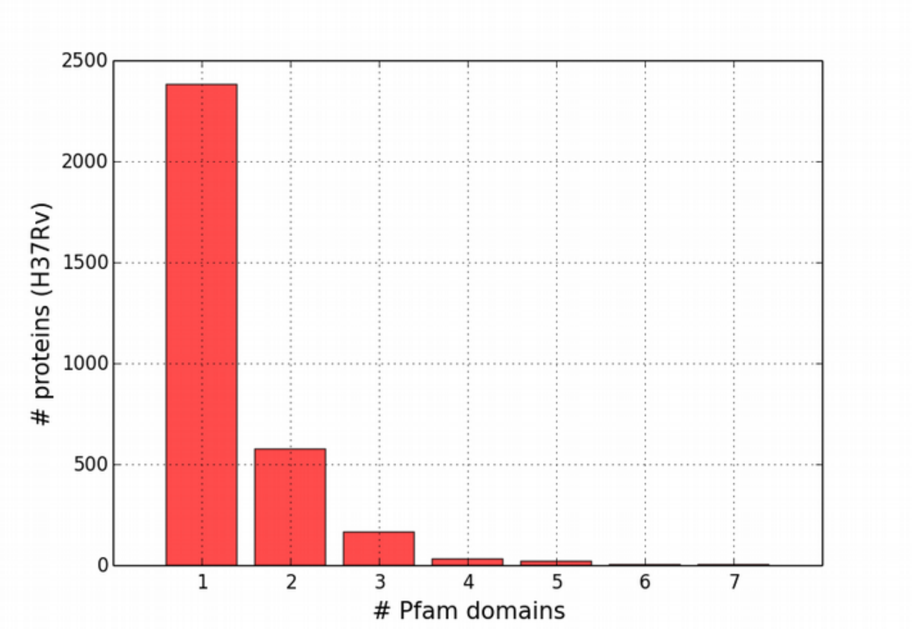

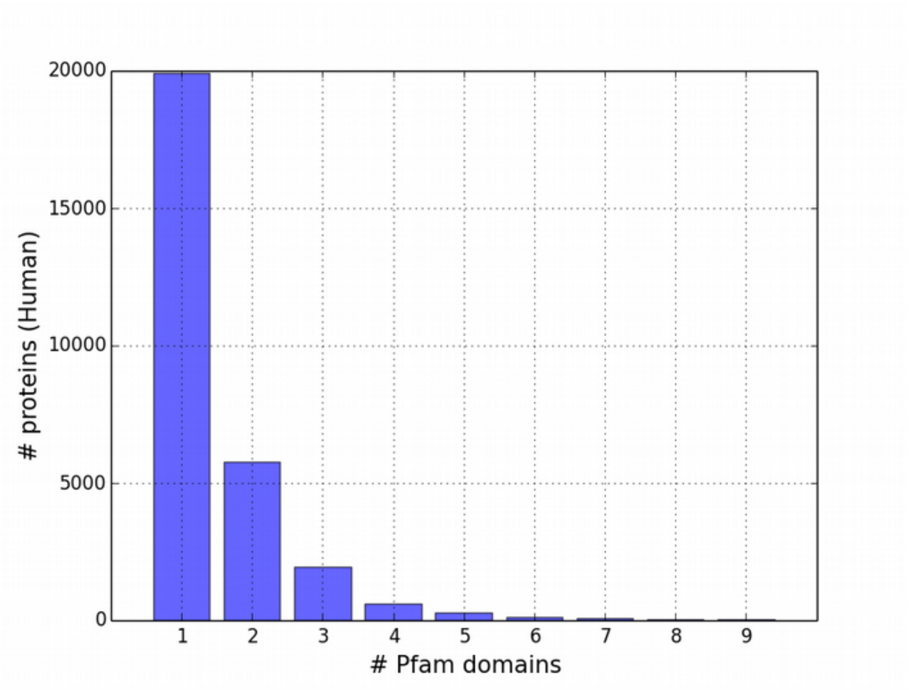

Fig. S1. **Histograms summarizing the occurrences of Pfam-A functional domains in the human (blue) and MTB (red) proteomes.** Nearly 30 % of the proteins in both species have been assigned more than one domain.

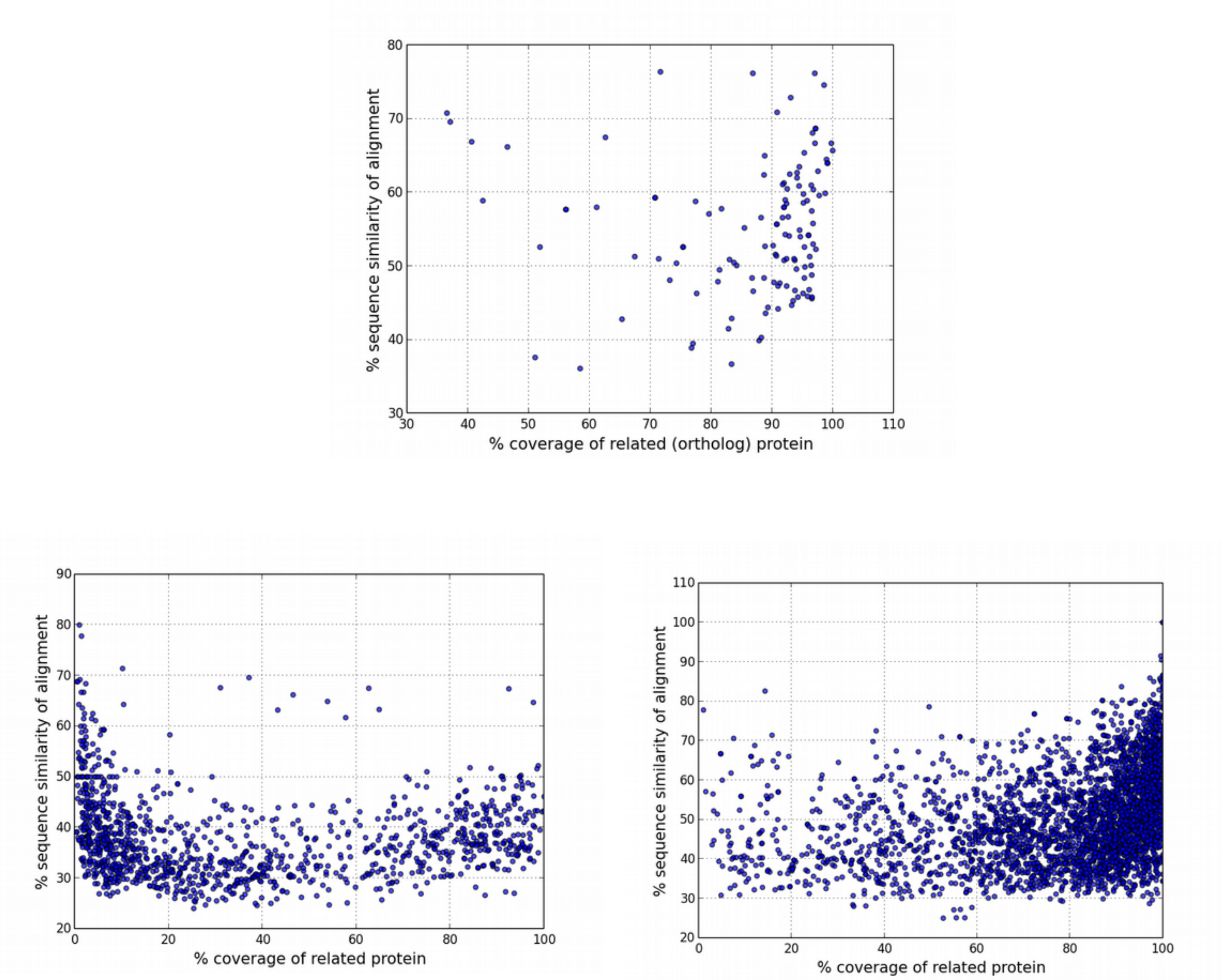

Fig S2. **Pair-wise comparisons between MTB proteins and their putative orthologs listed in the Integr8 (upper figure), eggNOG (lower left) and KEGG Orthology/KO (lower right) databases.** Note the relatively smaller number of orthologs assigned by Integr8.

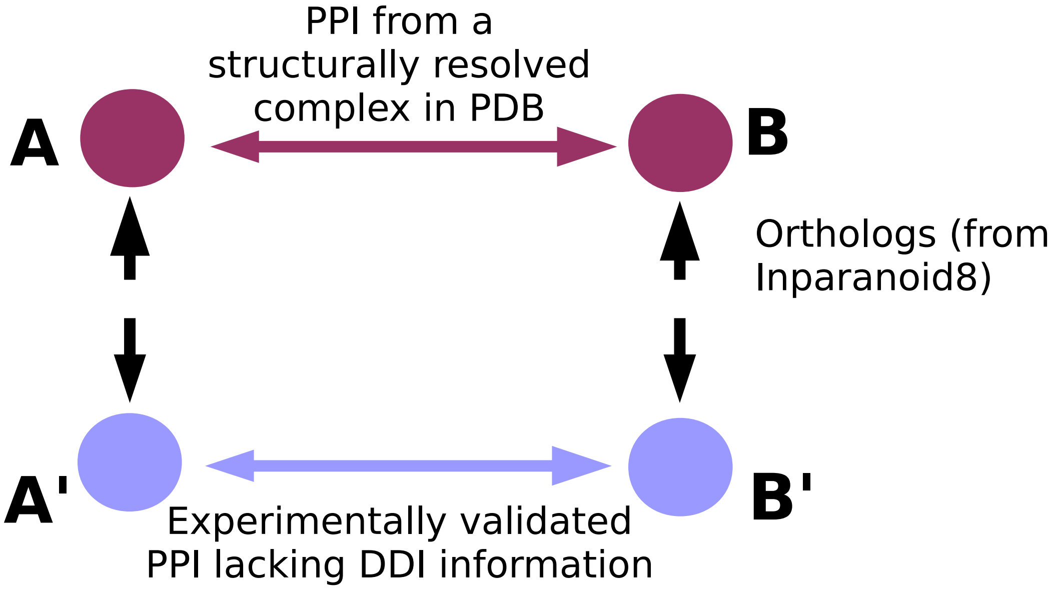

Fig S3. **Using the interolog approach to extend the limited set of DDI templates provided by PDB.** Evolutionarily conserved PPIs (involving orthologs) are assumed to share a common underlying pattern of domain-domain interactions. This allows interacting domains to be inferred for PPIs for which structure information is not directly available (here, A'-B').

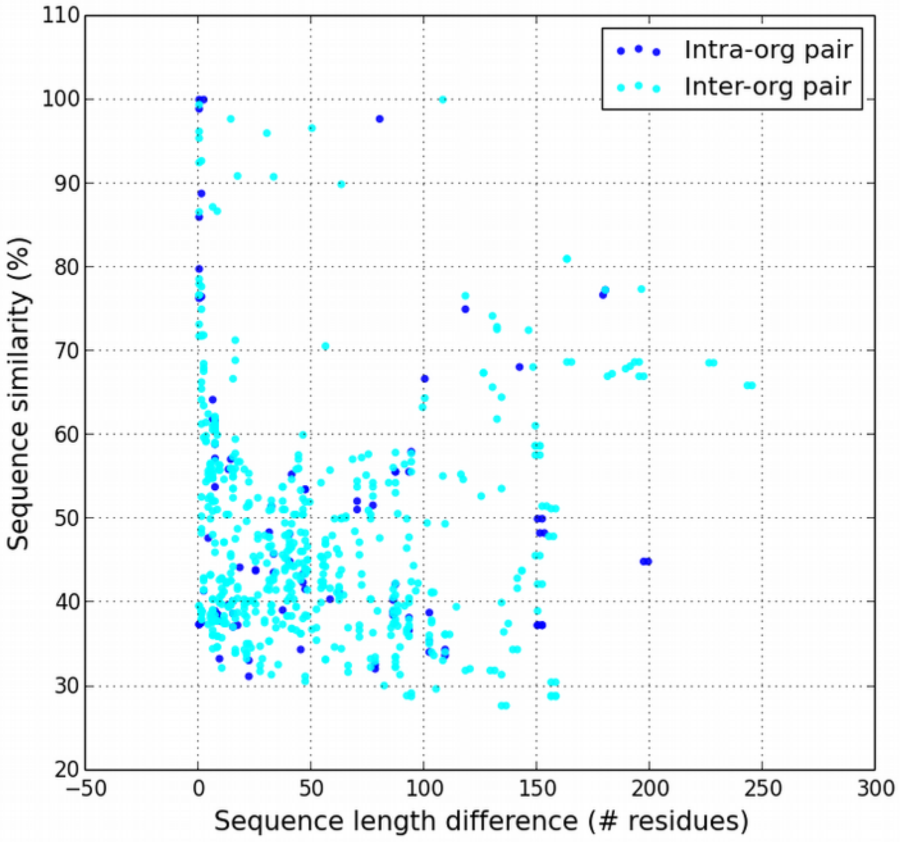

Fig S4. **Diversity in terms of length and sequence composition across the polypeptide sequences assigned to the Ulp1 protease family C-terminal catalytic domain (PF02902).** The scatter plot follows from all-vs-all pairwise sequence alignment using the Smith-Waterman method. Pairs coming from the same proteome (blue) or from different proteomes (cyan) have been assigned different colors.

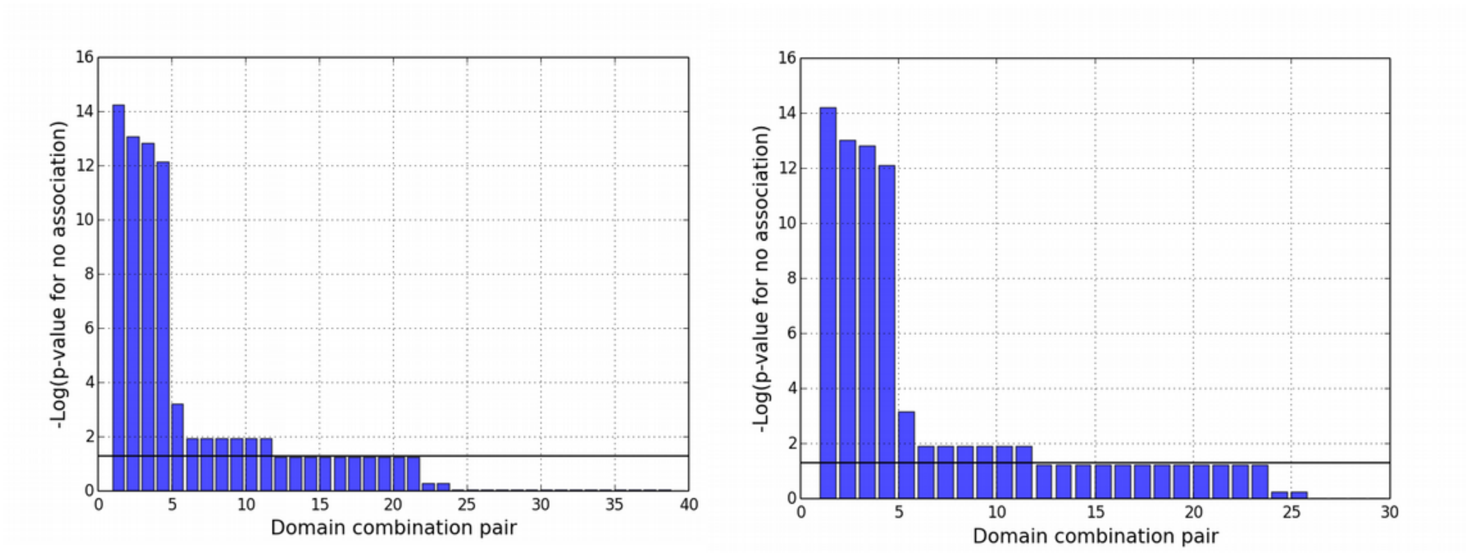

Fig S5. **Statistical over-representation of interacting Pfam domain pairs (from iPfam/3DID) in a PPI network for co-localized, coexpressed cytosolic proteins in *E. coli.*** Several domain pairs do not show significant association with the PPI set. The darker horizontal line represents the p-value < 0.05 threshold.
