# Supplementary material for: Using structural knowledge in the protein data bank to inform the search for potential host-microbe protein interactions in sequence space: application to Mycobacterium tuberculosis
Source: BMC Bioinformatics. 2017 Apr 4;18:201. doi: 10.1186/s12859-017-1550-y (PMC5379762; doi:10.1186/s12859-017-1550-y)
Supplement: Supplementary file 6 — Summary of comparison of our predictions with those from four most recently published computational studies on host-MTB interactions. (PDF 49 kb) [file 12859_2017_1550_MOESM6_ESM.pdf]

Summary of comparison of our predictions with previous publications.

We considered the four most recently published computational studies on human-MTB interactions (refs. 20, 32, 33 & 70 in the main text). Of these, two publications (Rapanoel et al. 2013 [20] and Huo et al. 2015 [32]) did not include their full set of predictions, so no comparison could be made. Of the remaining two studies which have made their predictions available, we found no overlap with the predictions of Zhou et al. 2014 [33], but found about a dozen common predictions (at score threshold of 0.75) with the approach of Zhou et al. 2013 [70]. These protein pairs are listed below:

MTB ACC	Human ACC	MTB protein name(s)	Human protein name(s)
O05855	E7EQG2	rhlE/Rv3211	EIF4A2
O53645	P08183	Rv0194	ABCB1/MDR1/PGY1
O05855	P38919	rhlE/Rv3211	EIF4A3/DDX48/KIAA0111
O53639	Q9UJ83	oxcA/Rv0118c	HACL1/HPCL/HPCL2/PHYH2/HSPC279
O05855	Q96A72	rhlE/Rv3211	MAGOHB/MAGOH2
P96890	P11498	accA3/Rv3285	PC
O05855	P60842	rhlE/Rv3211	EIF4A1/DDX2A/EIF4A
O05855	O15234	rhlE/Rv3211	CASC3/MLN51
O05855	Q9HCG8	rhlE/Rv3211	CWC22/KIAA1604/NCM
O05855	P61326	rhlE/Rv3211	MAGOH/MAGOHA
O05855	Q14240	rhlE/Rv3211	EIF4A2/DDX2B/EIF4F
O05855	J3KT12	rhlE/Rv3211	EIF4A1

Limited overlap with these earlier studies may be attributed to differences in the background PPI/DDI databases used, besides differences in the details of the methodologies followed.
